# Spatial variation in macrobenthic assemblages and their relationship with environmental factors in the upstream and midstream regions of the Heihe River Basin, China

**DOI:** 10.1007/s10661-020-08822-0

**Published:** 2021-01-11

**Authors:** Yu Wang, Juan-Juan Liu, Wei Liu, Qi Feng, Bao-long Li, Han Lu, Shuang Wang

**Affiliations:** 1 College of Energy and Power Engineering, Lanzhou University of Technology, Lanzhou, 730050 China; 2 Key Laboratory of Ecohydrology of Inland River Basin, Northwest Institute of Eco-Environment and Resources, Chinese Academy of Sciences, Lanzhou, 730000 China

**Keywords:** Macrobenthic organisms, Spatial distribution, Environmental variables, Aquatic ecologic, Heihe River Basin

## Abstract

The Heihe River is a typical inland river under increasing anthropogenic pressure. To explore the characteristics of the macrobenthic assemblages and their relationships with environmental factors in the upstream and midstream regions of this basin, abiotic conditions and macrobenthic assemblages were investigated in the summers of 2018 and 2019. A total of 50 species were collected, and Arthropoda and mollusks were the dominant groups. A significant increase in standing stock was observed from the upstream to midstream, and predators (PR) were the main functional feeding group. A one-way analysis of variance (ANOVA) revealed that the Shannon-Wiener index and Margalef’s index values significantly differed at the spatial scale (*P* < 0.05). A redundancy analysis (RDA) and Pearson correlation analysis showed that the spatial heterogeneity of the macrobenthos was influenced by the biochemical oxygen demand (BOD_5_), water temperature (WT), total nitrogen (TN), salinity, electrical conductivity (EC), total dissolved solids (TDS), dissolved oxygen (DO), and potassium permanganate index (COD_Mn_) (*P* < 0.05). The spatial variation of macrobenthos was mainly governed by natural conditions and human disturbances.

## Introduction

Rivers are important carriers of freshwater resources that maintain the biosphere water cycle, regulate nutrient migration and accumulation, and promote energy balance, and they also play a major role in hydrological conditions and sustainable ecological development (Gelwick 2000; Xu 2017; Pukšec et al. 2019). Rivers experience a gradient of environmental conditions caused by natural variables, such as climate, topography, and geology, and the intensification of anthropogenic activities caused by cascade hydropower development and industrial and agricultural construction. These activities generate various land-based pollutants that are ultimately discharged into the adjacent river water. The natural ecological processes and dynamic balance of the original river are forced to change as a consequence, which impacts the material, energy, and flow transport of the river channel to a large extent and affects the water dynamic conditions, hydrological processes, and medium transport mechanisms of the river. Environmental factors can reflect river ecological process changes that lead to the succession of river biodiversity and affect the distribution of macrobenthos communities (Hupp and Simon 1991; Frost et al. 1999; Dong 2003; Wen et al. 2018).

Among the biological components of river ecosystems, macrobenthic organisms are essential for ecosystem functioning due to their diverse feeding habits and ability to adapt to different environmental conditions. These organisms play a critical role in the energy flow and material cycling of the benthic system by serving as food for a variety of PRs; thus, they can greatly influence the species composition and abundance of tertiary consumers (Currie and Small 2005; Mandal and Harkantra 2013). Compared with other taxonomic groups (e.g., fish and algae), benthic organisms act as an important medium for maintaining ecosystem functions by accelerating the decomposition of organic detritus and regulating the exchange of material at the mud-water interface and the self-purification of water bodies (Covich et al. 2004; Rabení et al. 2005). This community is critically linked to material circulation and energy flow and thus is important for understanding the structure, function, and health status of river ecosystems (Devine and Vanni 2002). Macrobenthic organisms are slow-moving and have a relatively fixed activity range, long life span, and stable living habits, and the diverse species composing the macrobenthos are particularly sensitive to environmental perturbations and easily collected (Peng et al. 2014). Due to these unique biological properties, macrobenthic organisms are used as effective ecological indicators to evaluate benthic health (Tong et al. 2013; Keeley et al. 2014). Environmental factors play essential roles in the growth, reproduction, and community succession of macrobenthic organisms. Therefore, investigations of the response relationship between macrobenthic organisms and the water environment are important because they provide powerful information for explaining the cumulative effect and have guiding significance for understanding material cycling, energy flow, and information transmission in aquatic ecosystems and improving strategies for addressing the ecological protection and restoration of watersheds.

Macrobenthos are a key link of ecosystem dynamics and crucially important for regulating or modifying the physicochemical and biological evolution of the whole aquatic ecosystem. The research methods and biological indicators of macrobenthic community characteristics and ecological effects are also different. The Shannon-Wiener diversity index (Shannon and Weaver 1963), Simpson’s diversity index (Simpson 1949), Margalef’s species richness index (Margalef 1957), and Pielou’s evenness index (Pielou 1966) are widely used in studies of the community structure and diversity of macrobenthos. These indicators are mainly used to compare the changes of species composition between damaged and reference communities, thus reflecting the evolution characteristics of different community structures and representing environmental monitoring or aquatic ecological health status indicators (Stevenson 1984; Metcalfe 1989; Shokri et al. 2014). In recent years, research has primarily focused on the response relationship between macrobenthic organisms and environmental parameters. Simultaneously determining various environmental factors is used to reveal the deeper interactions, and such work is usually accomplished via the redundancy analysis (RDA) and canonical correspondence analysis (CCA) methods. In addition, with the introduction of multivariate statistical methods, more in-depth quantitative studies of the microbenthic community structure have been performed. The Pearson or Spearman correlation matrix analysis of macrobenthic organisms and environmental factors is carried out using SPSS software to further verify the stability of the community structure and determine the driving factors that affect the community structure and biodiversity.

Cai et al. (2010) showed that Margalef’s species richness index and Pielou’s evenness index were significantly negatively correlated with the trophic state index, which indicated that the macrobenthos community structure tended to be simplified as the nutrient level increased. Yan et al. (2017) measured long-series variation characteristics of macrobenthos based on the Shannon-Wiener diversity index, Simpson’s diversity index, Margalef’s species richness index, and Pielou’s evenness index and revealed that the water temperature (WT), salinity, and depth were the main driving factors affecting the spatial sequence change of macrobenthic organisms based on the CCA and RDA methods. Liu et al. (2016) explored the spatiotemporal heterogeneity of macrobenthos by the Shannon-Wiener diversity index, Simpson’s diversity index, Margalef’s species richness index, and Pielou’s evenness index and determined that the WT, pH, total nitrogen (TN), and heavy metal gradient changes (cadmium: Cd; lead: Pb; mercury: Hg) in sediments were the crucial factors impacting the spatial and seasonal fluctuations of macrobenthos; additionally, the habitat and spatial distribution of macrobenthos were also disturbed by gate and dam operations, slope consolidation, shore vegetation belts, and sand mining activities. Buss et al. (2002) clarified that the spatiotemporal variability of macrobenthic assemblages could be determined by the Shannon-Wiener diversity index and Pielou’s evenness index while the important influence of dissolved oxygen (DO), chloride, and environmental degradation on the macrobenthos distribution could be determined by the CCA. Li et al. (2012) found that the multiple spatial orders of macrobenthic organisms were determined by latitude, forest coverage, shoal habitat, silt layer, and temperature based on the CCA method. Feld and Hering (2007) used the CCA and RDA methods and found that watershed-scale landscape characteristics and hydrological factors explained 11.4%, 22.1%, and 15.8% of the spatial variation of macrobenthos at the watershed, river reach, and point scales, respectively. Li et al. (2015b) concluded that human activity pressure factors (pH, TN, potassium permanganate index (COD_Mn_), electrical conductivity (EC), total dissolved solids (TDS), ammonia nitrogen (NH_3_-N), hardness and habitat quality) had the most significant impact on the spatial heterogeneity of macrobenthos based on the RDA method and Pearson’s correlation matrix analysis and environmental factors at different scales had synergistic effects on macrobenthic organisms.

The Heihe River Basin (HRB) is located in the central part of the Hexi Corridor in the arid region of northwestern China, and it is the second largest inland river basin in China. Regarding the competition for water between the economy and the ecosystem, the HRB is considered representative of all inland river basins around the world, including the Aral Sea Basin (Feng et al. 2001). In recent years, severe deterioration of the water and ecological environment of the HRB has occurred, especially in the upstream and midstream regions (Cheng et al. 2014). This deterioration in the upstream area has largely been caused by local anthropogenic activities, including deforestation, overgrazing, grassland reclamation and cascade hydropower development. As a result, the continuity of the river ecosystem has been damaged and the physicochemical characteristics of the water body, medium transport patterns, and cumulative effects along the river have been greatly changed. Because of the population density in the midstream region, the quantity and quality of the water has been primarily affected by industrial and agricultural sewage and excessive development of oases, and these changes have had a series of impacts on the water environment system succession and river ecological health (Feng et al. 2001; Chen et al. 2004; Cheng et al. 2014; Hao et al. 2014). As a result, many natural oases have disappeared and the amount of water entering the downstream area has significantly decreased. These changes have led to prominent ecological problems, such as the simplification of habitat, decreases in biodiversity, and declines in ecological function (Burford et al. 2007; Feld and Hering 2007; Li et al. 2015b). Thus, evaluating the relationship between macrobenthos and environmental parameters is of critical importance in the HRB.

A previous study addressed the spatial variation in the macrobenthic assemblages in the HRB by comparing the results obtained in historical surveys from the literature with those from a field investigation (Li et al. 2001). The authors concluded that the microbenthic faunal assemblages changed greatly over time due to natural environmental variation and human disturbances. However, studies of long-term changes in the macrobenthos community of the HRB are lacking, with most studies focusing on the response of phytoplankton and zooplankton to water ecological health (Li et al. 2000; Hao et al. 2014). Based on the typical upstream and midstream sections of the HRB, the present study aimed to (1) analyze the spatial variation in water environmental parameters and evaluate the benthic environmental health, (2) measure the variation in macrobenthic assemblages over a large spatial scale, and (3) explore the relationships between microbenthic faunal assemblages and environmental factors. To achieve these aims, we performed a systematic ecological investigation of the water environment and macrobenthos in the upstream and midstream sections of the HRB and analyzed the survey data via a series of statistical methods to provide a theoretical basis for ecological management and scientific protection of the HRB.

## Materials and methods

### Study area

**Fig. 1:**
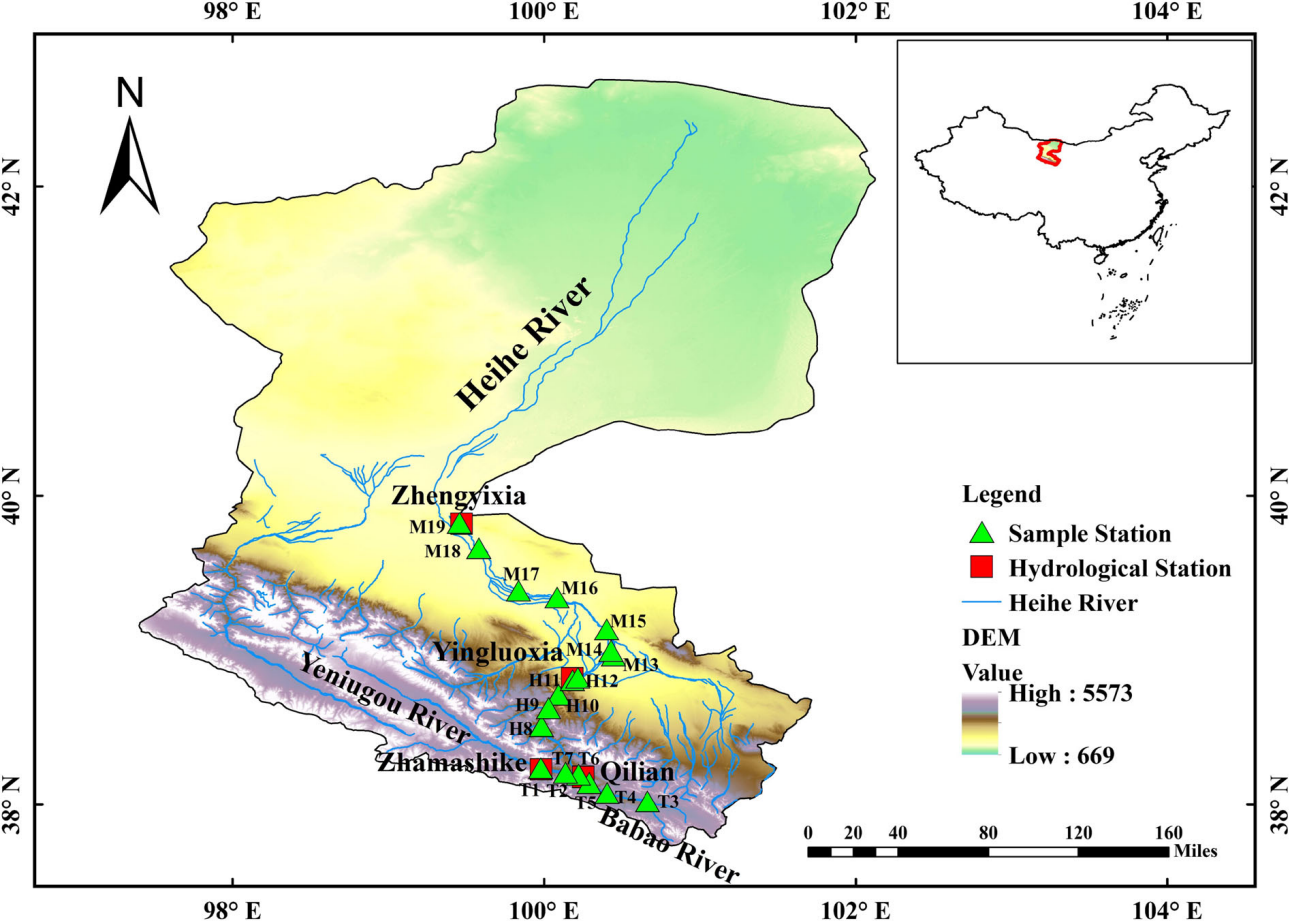
Map of the study area showing the sampling stations

The Heihe River (96° 42′−102° 04′ E, 37° 45′−42° 40′ N) is the second largest inland river in the arid region of Northwest China, and it originates in the northern foot of the Qilian Mountains. It has a drainage area of 14.3 × 10^4^ km^2^, and the total length of the mainstream is approximately 821 km. The river consists of three parts, namely the upper mountainous area (the source of the river), the middle oasis area (incorporating towns such as Zhangye and Jiuquan), and the lower terminal arid area near Ejina. In this study, the upstream and midstream areas of the HRB were selected as the study area (Fig. 1). The upstream area, with an elevation of 2000−5000 m, is the water conservation area in the Qilian Mountains and has a mean annual temperature of − 3 to 4 °C. At elevations above 4000 m, the vegetation is very sparse and dominated by cushion plants. Meadows and shrubs occur below 3300 m. The mean annual precipitation is greater than 350 mm, and the mean annual water resource availability is 1.6×10^9^ m^3^. Eight cascade hydropower stations were developed successively, thus creating the main runoff-producing area in the HRB. The cultivated oasis area in the midstream region is dominated by irrigated farmland and rich in light and heat resources. The mean annual temperature in this subbasin is approximately 3−7 °C; the mean annual precipitation ranges from 50 to 150 mm; and the mean potential evaporation rate is approximately 1400 mm; thus, this area is the main utilization area of Heihe River resources (Chen et al. 2006). As a typical inland river, the Heihe River is supplied by surface runoff, ice and snow meltwater, and groundwater formed by precipitation, among which atmospheric precipitation (90%) is the main source (Chen et al. 2006; Yang et al. 2011).

### Sampling stations

To determine the response of the macrobenthic assemblages in the Heihe River to water environmental factors, we selected 19 stations to be representative of three zones in the upper-middle reaches basis on the physical and geographical characteristics, cascade hydropower operations, and industrial and agricultural development (Fig. 1). Six sampling points (stations T1 to T6) were established in the upstream tributary area according to the distribution of animal husbandry and enterprise operating conditions, six sampling points (stations H7 to H12) were established in the upstream area of the main stream on the basis of cascade hydropower construction, and seven sampling points (stations M13 to M19) were established in the midstream according to industrial and agricultural construction and administrative division conditions. Among the three regions, those with relatively little disturbance resulting from human activities were located in the upstream region of the Heihe River.

### Macrobenthos sampling

Samples for the evaluation of the macrobenthos assemblage and water quality in the upstream and midstream regions of the HRB were collected in August 2018 and July 2019. Macrobenthos organisms were collected with a combination of quantitative and qualitative methods, with a Peterson dredger (1/16 m^2^) used for quantitative collection and hand-dip nets used for qualitative collection. The samples were collected repeatedly 2 to 3 times at different positions at each sampling point, and the mean value was used. The collected samples were filtered and washed with a 60-mesh screen and then sorted at the site. The sorted samples were preserved in 4−10% formalin and transported to the laboratory for further analysis. Sorting of the samples was performed in the laboratory, and 75% ethanol was used to fix the clean macrobenthos (oligochaetes were preserved with formalin to prevent breakage).

The macrobenthic organisms were identified to the species level and classified using the relevant identification guides and then counted and weighed (Liu 1979; Morse et al. 1994; Liu 1999; Peter and Dudgeon, 2001). All the macrobenthic samples were identified to at least the genus level and assessed in terms of their distribution, abundance, and diversity.

The samples were divided into 6 functional feeding groups (FFGs): shredders (SH), collector-filterers (FC), collector-gatherers (GC), scrapers (SC), predators (PR), and omnivores (OM) (Cummins and Klug 1979). The species were also classified into 3 categories based on their pollution tolerance value (*X*) (Wang 2003): pollution-tolerant species (*X* ≥ 7), moderately tolerant species (3 < *X* < 7), and sensitive species (*X* ≤ 3).

### Environmental variables

Samples of surface water were collected with 1-L prelabeled plastic containers at each study station. For the determination of water environmental factors, the WT, pH, EC, TDS, DO, and salinity were directly measured in the field. WT was estimated at each sampling station using a digital display thermometer (model XMD200; precision, 0.1 °C), and the pH, EC, TDS, DO, and salinity were determined on-site using a HACH (model DR300) portable water quality analyzer. Additional 1-L water samples were collected and fixed after storage in a 4 °C incubator in the laboratory to determine the TN, total phosphorus (TP), NH_3_-N, chemical oxygen demand (COD_cr_), and COD_Mn_. Water chemical indicators, such as TN, were determined via alkaline potassium persulfate digestion and UV spectrophotometry (GB11894 - 1989), whereas TP was measured by colorimetry (GB11893 - 1989). NH_3_-N was determined using Nessler’s reagent method (GB7479 - 87). COD_cr_ and COD_Mn_ were estimated using the acidic potassium permanganate method (GB/T11892 - 1989). Water samples used in the biochemical oxygen demand (BOD_5_) analysis were collected in 250 mL dissolved oxygen bottles and incubated in the dark for five days for the measurement of BOD_5_ referring to the “Water and Wastewater Monitoring and Analysis Methods (4th Edition)” of the State Environmental Protection Bureau of China (State Environment Protection Bureau of China 2002). Each sample was measured three times, and the average value was used.

### Data analyses

The biological properties of each sampling site included the macrobenthos biomass (g/m^2^), density (ind./m^2^), species number (*S*), Shannon-Wiener diversity index (*H*′) (Shannon and Weaver 1963), Margalef’s species richness index (*d*_M_) (Margalef 1957), Pielou’s evenness index (*J*) (Pielou 1966) and dominance index (*Y*) (Chen and Wang 1995). The three biodiversity indexes were calculated according to Eq. (1) to (3), and the dominance index was calculated using Eq. (4). Data from the same station collected during two different cruises were averaged for every period.

1 $$ H=-\sum \limits_{i=1}^s\left({n}_i/N\right){\log}_2\left({n}_i/N\right) $$

2 $$ {d}_M=\left(S-1\right)/1 nN $$

3 $$ J=\left(-\sum \limits_{i=1}^s\left({n}_i/N\right){\log}_2\left({n}_i/N\right)\right)/{\log}_2S $$

4 $$ Y=\left({n}_i/N\right)\times {f}_i $$

where *N* is the total number of individuals, *n*_*i*_ is the number of individuals of the ith species, *f*_*i*_ is the frequency of occurrence of the ith species, and *S* is the number of individuals of macrobenthic species. When *Y* > 0.02, a species is considered a dominant species (McNaughton 1967; Chen and Wang 1995).

The sampling plots in the HRB were drawn using ArcGIS (version 10.4, USA). The abundance of macrobenthic assemblages was compared to identify significant variations across the different zones using a one-way analysis of variance (ANOVA). Canonical ordination was used to reveal the relative importance of environmental variables in determining the structural composition among macrozoobenthic organisms. CCA and RDA were used to investigate the biological-environmental relationships after performing a detrended correspondence analysis (DCA) to determine whether to use CCA or RDA (Feld and Hering 2007). Based on the DCA, if the maximum gradient length of the axes was greater than 4 SD, then the CCA was more suitable, while if the maximum gradient length of the axes was less than 3 SD, then the RDA was more suitable (Leps and Smilauer 2003). The RDA was used to assess the correlations between the macrobenthic organisms and environmental parameters, because in the preliminary DCA, the maximum gradient length of the axes was 2.53 SD. In the RDA, forward selection analyses and Monte Carlo permutation tests were performed to identify the important environmental parameters that influence the abundance and distribution of the macrobenthos. Before the statistical analyses, the data were log_10_(*x* + 1) transformed to reduce the heterogeneity of variance (all environmental parameters except for pH), and then a Pearson correlation analysis was performed to evaluate the relationship between them. Microsoft Excel (version 2010, USA), ArcGIS (version 10.4, USA), IBM SPSS Statistics (version 20.0, USA), OriginPro (version 9.0, USA), and CANOCO V5.0 software were used for the data analyses.

## Results

### Environmental parameters

Descriptive statistics regarding the 12 physicochemical indexes evaluated for the 19 sampling stations located in the upstream and midstream regions of the HRB are presented in Table 1. The ANOVA demonstrated that the environmental variables in the HRB showed significant variability in the different zones (Table 1). The WT during the monitoring period ranged between 11.05 and 29.35 °C and gradually increased from the upstream to midstream, which showed significant differences (*P* < 0.05). The pH value mainly fell between 8.76 and 9.10, with the water being weakly alkaline. The EC, TDS, and salinity were 479 to 873 μS/cm, 232 to 428 mg/L, and 0.23 to 0.43‰, respectively. These three indicators presented consistent variations along the river, with the highest values in the middle reaches and significant differences observed between the upstream and midstream regions (*P* < 0.05). On the physical level, the three indicators are related to and complement one another. A greater content of dissolved substances in the water body corresponded to better conductivity and higher salinity (Han et al. 2009), which was confirmed in this study.

**Table 1:** Statistical analysis of different water environmental parameters (mean ± SD). The same line labelled by different letters in superscripts of the table indicated relevant significant differences (*P* < 0.05). *WT*: water temperature; pH, *EC*: electrical conductivity; *TDS*: total dissolved particle; *DO*: dissolved oxygen; salinity; *TP*: total phosphorus; *TN*: total nitrogen; *NH*_*3-*_*N*: ammonia nitrogen; *BOD*_*5*_: biochemical oxygen demand; *COD*_*c*r_: chemical oxygen demand; *COD*_*Mn*_: potassium permanganate index

Variable	Upper tributary(*n* = 6)	Upper mainstream(*n* = 6)	Middle stream(*n* = 7)	Measured range(*n* = 19)	*F*	*P*
WT(°C)	14.68±3.28^b^	17.04±1.26^b^	23.81±3.18^a^	11.05~29.35	19.36	< 0.05
pH value	8.98±0.05^a^	8.95±0.14^a^	9.04±0.06^a^	8.76~9.10	1.93	0.178
EC(μS/cm^1^)	638.50±81.71^ab^	566.50±74.90^b^	703.86±118.78^a^	479~873	3.34	0.061
TDS(mg/L^1^)	313.67±42.26^ab^	275.17±37.26^b^	343.79±59.57^a^	232~428	3.28	0.064
DO(mg/L^1^)	7.53±0.59^a^	7.29±0.94^a^	7.84±0.63^a^	5.72~9.00	0.92	0.418
Salinity(‰)	0.32±0.04^ab^	0.28±0.04^b^	0.35±0.06^a^	0.23~0.43	3.71	0.047
TP(mg/L^1^)	0.09±0.07^b^	0.20±0.10^a^	0.16±0.08^ab^	0.01~0.03	2.60	0.105
TN(mg/L^1^)	0.70±0.44^b^	1.33±0.45^a^	1.73±0.55^ab^	0.42~2.38	7.32	0.006
NH_3-_N(mg/L^1^)	0.14±0.06^a^	0.13±0.04^a^	0.13±0.09^a^	0.05~0.30	0.07	0.931
BOD_5_(mg/L^1^)	0.33±0.22^b^	0.90±0.68^ab^	1.29±0.50^a^	0.14~2.20	5.95	0.012
COD_Cr_(mg/L^1^)	15.75±4.9^a^	15.13±2.45^a^	13.69±4.64^a^	7.12~22.94	0.42	0.666
COD_Mn_(mg/L^1^)	2.43±0.41^b^	3.05±0.43^a^	2.02±0.49^b^	1.41~3.74	8.58	0.003

The mean DO, TN, TP, NH_3_-N, BOD_5_, COD_cr_, and COD_Mn_ values were 7.56 mg/L, 1.28 mg/L, 0.15 mg/L, 0.13 mg/L, 0.87 mg/L, 14.80 mg/L, and 2.48 mg/L, respectively. The “GB/T3838-2002 Environmental Quality Standards for Surface Water” (GB/T3838 - 2002) indicated that NH_3_-N, BOD_5_ and COD_cr_ belonged to class I, COD_Mn_ belonged to class II, and TP belonged to class III, which basically met the standard limits of the functional zone; however, TN exceeded the standard limit of class III and reached class IV. The measured range showed that the upstream concentration of COD_cr_ was significantly higher than that in the middle reaches, and it exceeded the class III standard of the functional zone in the upstream tributary station T5 and the middle mainstream station M19. The concentration of TP exceeded the water standard of class III by 1.62 times in the functional area at T4, H8 to M13, and M17 to M18, and it even reached class V in some river sections, indicating that the water was seriously polluted. The midstream concentration of TN was higher than that of the upstream, and the difference was significant (*P* < 0.05). The TN concentration at T4 in the upstream tributary exceeded class III, although the values were lower at H8 in the upper mainstream and M14 in the middle mainstream, and the maximum value of TN exceeded the water standard of class III by 2.39 times. The water pollution in most river sections was severe, and the water had a high nutrient concentration.

The Pearson correlation analysis (Table 2) showed that significant correlations occurred among EC, TDS, salinity, and TN, indicating that their sources were consistent and similar. High positive correlations were observed between BOD_5_ and pH, DO, and TN, indicating that the BOD_5_ increased significantly with increasing pH, DO, and TN. The correlations between WT and EC, TDS, salinity, TN, and COD_Mn_ were strong, which indicated that WT had an important impact on water quality.

**Table 2:** Pearson correlation analysis of water environmental parameters. *: Indicating significant correlation at 0.05 level (*p* < 0.05); * *: Indicating significant correlation at 0.01 level (*p* < 0.001). *WT*: water temperature; pH, *EC*: electrical conductivity; *TDS*: total dissolved particle; *DO*: dissolved oxygen; salinity; *TP*: total phosphorus; *TN*: total nitrogen; *NH*_*3-*_*N*: ammonia nitrogen; *BOD*_*5*_: biochemical oxygen demand; *COD*_*cr*_: chemical oxygen demand; *COD*_*Mn*_: potassium permanganate index

	WT	pH value	EC	TDS	DO	Salinity	TP	TN	NH_3_-N	BOD_5_	COD_cr_	COD_Mn_
WT	1											
pH value	0.179	1										
EC	0.489*	0.150	1									
TDS	0.485*	0.142	0.999**	1								
DO	0.090	0.436	0.153	0.152	1							
Salinity	0.483*	0.165	− 0.998**	0.998**	0.143	1						
TP	0.442	0.331	0.223	0.224	0.038	0.212	1					
TN	0.714**	− 0.108	0.465*	0.457*	0.299	0.432	0.271	1				
NH_3_-N	− 0.063	− 0.046	0.332	0.331	− 0.160	0.353	− 0.057	− 0.076	1			
BOD_5_	0.400	0.541*	0.287	0.268	0.480*	0.281	0.354	0.506*	0.018	1		
COD_cr_	− 0.315	− 0.171	0.316	0.307	0.107	0.302	− 0.128	0.027	0.326	− 0.035	1	
COD_Mn_	− 0.564*	− 0.196	− 0.344	− 0.346	− 0.022	− 0.350	0.155	− 0.207	0.261	− 0.051	0.331	1

### Macrobenthic assemblages

#### Species composition and dominant macrobenthos species

During the study period, 50 species in total belonging to 3 phyla, 7 classes, 15 orders, and 32 families were collected and identified from the upstream and midstream regions of the HRB (Table 3), including 37 species (accounting for 74% of the total *S*) of arthropods that belonged to 3 classes, 10 orders, and 24 families; 11 species (accounting for 22% of the total *S*) of mollusks that belonged to 2 classes, 3 orders, and 6 families; and 2 species of annelids (accounting for 4% of the total *S*) that belonged to 2 classes, 2 orders, and 2 families.

**Table 3:** Community structure of macrobenthos in upstream and midstream of HRB

Phylum	Class	Order	Families	Genera	Species	Proportion (%)
Arthropoda	Insecta	Diptera	3	3	3	74
		Odonata	5	6	8	
		Trichoptera	2	2	2	
		Plecoptera	1	1	1	
		Hemiptera	4	4	5	
		Ephemeroptera	1	1	1	
		Coleoptera	5	10	14	
	Crustacea	Decapoda	1	1	1	
		Amphipoda	1	1	1	
	Arachnida	Araneae	1	1	1	
Mollusca	Gastropoda	Basommatophora	3	3	7	22
		Mesogastropoda	2	3	3	
	Lamellibranchia	Veneroida	1	1	1	
Annelida	Oligochaeta	Plesiopora	1	1	1	4
	Hirudinea	Rhynchobdellida	1	1	1	
Total	7	15	32	39	50	100

**Fig. 2:**
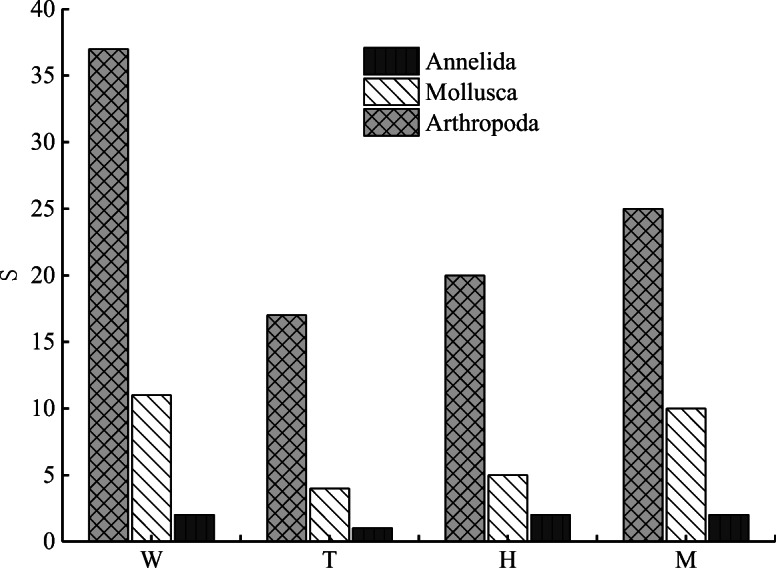
Number of macrobenthos various group species. The W is the whole study area, the T is upstream tributary, the H is upper main stream, and the M is middle stream

**Fig. 3:**
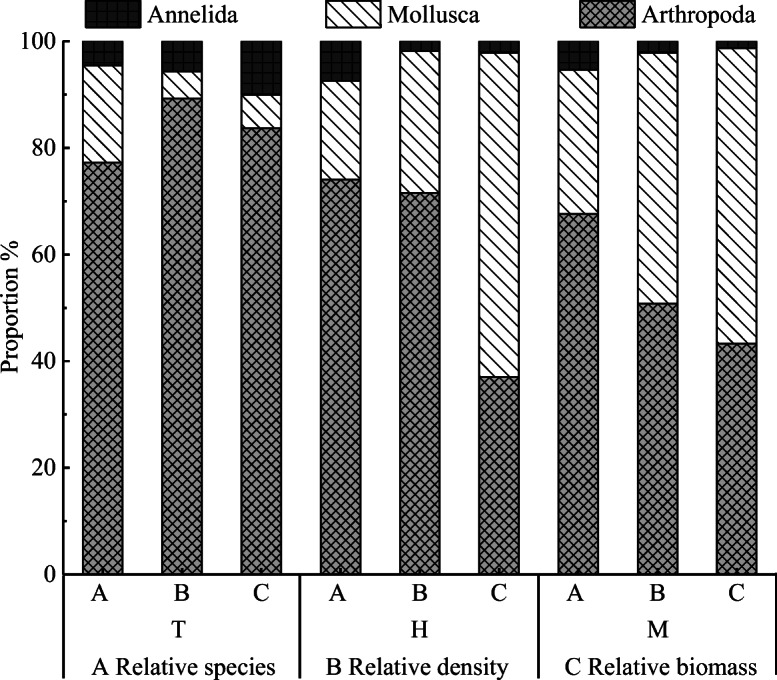
The relative number of species, the relative density and the relative biomass of different macrobenthos groups. The T is upstream tributary, the H is upper main stream, and the M is middle stream

**Fig. 4:**
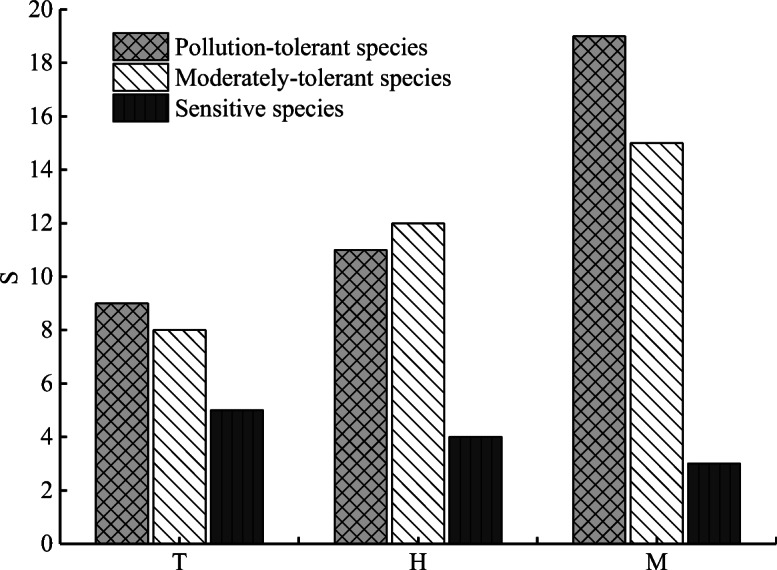
Number of species with different tolerance levels. The T is upstream tributaray, the H is upper main stream, and the M is middle stream

Arthropods were absolutely dominant and accounted for > 50% of the total species composition of the macrobenthic fauna in the upstream and midstream regions (Figs. 2 and 3). The spatial distribution of the species at the different points substantially varied, with an overall trend of the middle stream (37 species) > upper main stream (27 species) > upstream tributary (22 species). The number of species was largest (74%) in the midstream (ranged from 11 to 22 species), with a maximum reached at M13 (22 species). The *S* was lowest (varied from 2 to 10 species) in the upstream tributary (44%), with the minimum observed at T5 (2 species). The species distribution characteristics of macrobenthos with different degrees of tolerance in the upstream and midstream regions of the HRB are shown in Fig. 4. Pollution-tolerant species (19 species) and moderately tolerant species (15 species) occurred in the midstream, where the sensitive species were lowest in abundance (3 species); in contrast, the sensitive species were the most abundant (5 species) in the upstream tributary.

Dominant species of macrobenthos were identified at *Y* > 0.02. The spatial regional characteristics of the composition of dominant species significantly differed in the upstream and midstream regions of the HRB, where they were mainly dominated by arthropods, with mollusks included in some areas (Table 4). *Argyroneta aquatica* was the most dominant genus in the upstream and midstream regions of the HRB and appeared in every river section, with an occurrence rate of 100% and a dominance value ranging from 0.074 to 0.141. The dominant taxa of *Ischnura heterosticta* (0.041), *Radix auricularia* (0.033), *Gyraulus albus* (0.028), S*uecinea* sp. (0.025), *Palaemon modestus* (0.023), and *Chlaenius* sp. (0.022) were distributed throughout the study area. There were 5 dominant species in the upstream tributary, with a maximum frequency of 100% and a minimum frequency of 16.67%; 8 dominant species in the upper mainstream, with a maximum frequency of 100% and a minimum frequency of 33.33%; and 7 dominant species in the midstream, with a maximum frequency of 100% and a minimum frequency of 71.43%. The results showed that a greater number of dominant species and a smaller dominance value were related to a more complex and stable biological community structure (Chen et al. 2009).

**Table 4:** Dominant species and dominance degree of macrobenthos

Reach	Dominant species and dominance(*Y*)
The whole study area	Arthropoda: *Argyroneta aquatica* (0.11), *Ischnura heterosticta* (0.041), *Palaemon modestus* (0.023), *Chlaznius* sp. (0.022) Mollusca: *Radix auricularia* (0.033), *Cyraulus albus* (0.028), *Suecinea* sp. (0.025)
Upstream tributary	Arthropoda: *Argyroneta aquatica* (0.141), *Chlaznius* sp. (0.057), *Dolichus halensis* (0.04), *Tipulidae* (0.034), *Anisogammarus* sp. (0.033)
Upper mainstream	Arthropoda: *Tipulidae* (0.097), *Argyroneta aquatica* (0.074) *Chlaznius* sp. (0.071), *Baetis* sp. (0.053), *Ceraclea tsudai Akagi* (0.027), *Rhantus suturalis* (0.027) Mollusca: *Cyraulus albus* (0.063), *Radix auricularia* (0.034)
Middle stream	Arthropoda: *Argyroneta aquatica* (0.116), *Palaemon modestus* (0.092), *Ischnura heterosticta* (0.059), *Dragonfly larvae* (0.021) Mollusca: *Suecinea* sp. (0.118), *Radix auricularia* (0.067), *Cyraulus albus* (0.043)

#### Density and biomass of different groups of macrobenthos

**Fig. 5:**
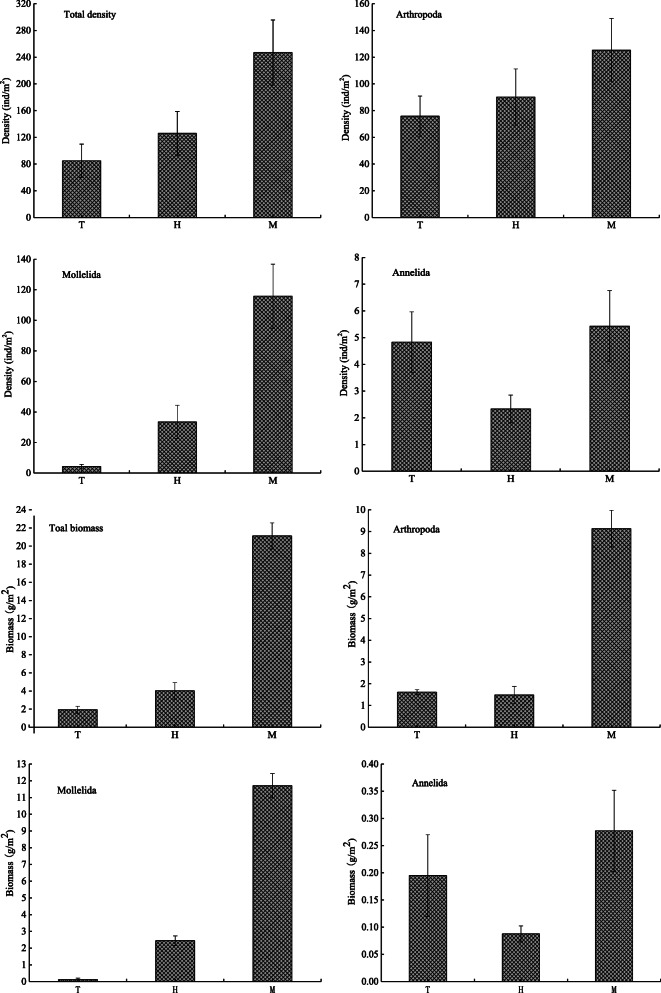
Density and biomass of different macrobenthos groups (mean ± SD). The T is upstream tributary, the H is upper main stream, and the M is middle stream

The spatial distribution characteristics of the mean density and biomass values of the different groups of macrobenthos in the upstream and midstream regions of the HRB are shown in Fig. 5. The results showed that the mean density of macrobenthos was 157.14 ind./m^2^ and the density at each sampling point ranged from 10 to 577 ind./m^2^. The mean biomass was 9.6613 g/m^2^, with a range of 0.0907 to 50.0562 g/m^2^. In terms of ecological groups, arthropods were the absolute dominant group of macrobenthos in this study area (Fig. 3), and their density (1876 ind./m^2^) accounted for 62.60% of the total density, followed by mollusks (1038 ind./m^2^, accounting for 34.69% of the total density) and annelids (81 ind./m^2^, 2.70%). In terms of biomass, mollusks accounted for the majority (97.3340 g/m^2^, accounting for 53.02% of the total biomass), followed by arthropods (82.5936 g/m^2^, 44.99%) and annelids (3.6374 g/m^2^, 1.98%). In terms of spatial changes, the existing stock of macrobenthos apparently increased from upstream to midstream. The mean total density and total biomass of the macrobenthos were 85 ind./m^2^ and 1.9365 g/m^2^ in the upstream tributary, respectively; 126 ind./m^2^ and 4.0193 g/m^2^ in the upper mainstream, respectively; and 247 ind./m^2^ and 21.1186 g/m^2^ in the middle stream, respectively. The ANOVA showed that there were significant differences between the upstream tributary and the main stream and middle stream (*P* < 0.05), while the difference between the upstream tributary and the main stream was not significant (*P* > 0.05).

The density and biomass of the different ecological groups of macrobenthos also significantly varied. The ANOVA results showed that there were significant differences in the total density and biomass of the macrobenthos, biomass of the arthropods, and density and biomass of the mollusks between the upstream and midstream regions of the HRB (*P* < 0.05); however, the density and biomass of the other groups did not significantly differ (*P* > 0.05). The total density and biomass of the arthropods and mollusks were significantly higher in the middle stream than the upper mainstream, while the density and biomass of annelids in the middle stream and upper main stream were much higher than those in the upstream tributary (Fig. 5). An ANOVA was performed to analyze the density and biomass of species with different tolerance levels (Table 5). The results showed that there were significant differences in the relative biomass of pollution-tolerant species (*F* = 5.150, *P* = 0.019), relative density of sensitive species (*F* = 5.554, *P* = 0.015) and relative biomass between the upstream and midstream regions of the HRB (*F* = 71.316, *P* < 0.05).

**Table 5:** Density and biomass characteristics of species with different tolerance levels (mean ± SD)

	Species	Upstream tributary(*n* = 6)	Upper mainstream(*n* = 6)	Middle stream(*n* = 7)	*F*	*P*
Proportion of density (%)	Pollution-tolerant species	2.83±3.78^b^	3.22±5.54^ab^	9.10±5.75^a^	3.086	0.074
	Moderately tolerant species	2.94±3.37^a^	5.31±8.07^a^	7.21±6.43^a^	0.749	0.489
	Sensitive species	2.65±1.72^b^	3.51±2.30^b^	9.01±5.56^a^	5.554	0.015
Proportion of biomass (%)	Pollution-tolerant species	0.67±0.83^b^	3.51±6.47^b^	10.70±7.46^a^	5.150	0.019
	Moderately tolerant species	1.70±1.77^a^	1.72±2.58^a^	11.36±15.46^a^	2.222	0.141
	Sensitive species	0.60±0.32^b^	0.79±0.55^b^	13.09±3.52^a^	71.316	< 0.05

#### Density and biomass of different functional feeding groups

**Fig. 6:**
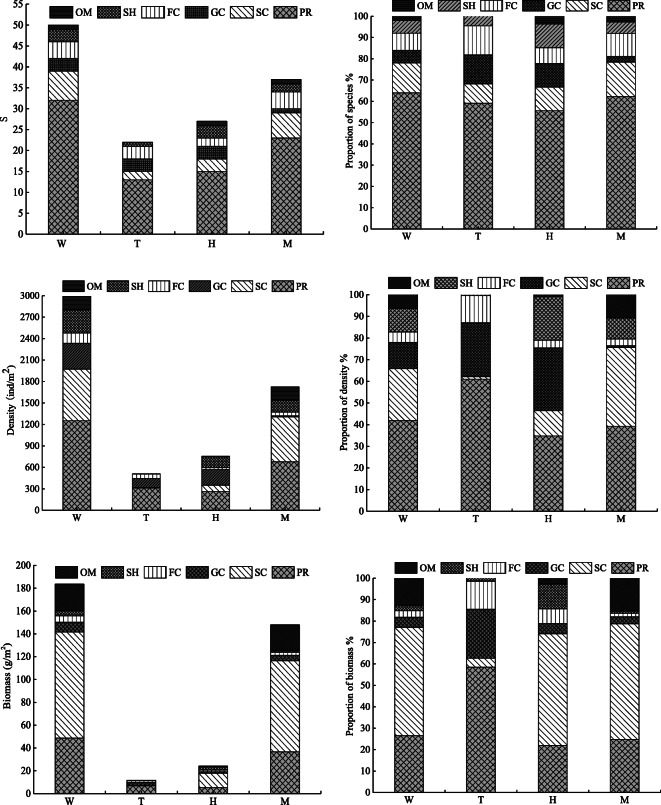
Species number, density and biomass of different functional feeding groups and their proportions in each reach. PR: predators; SC: scrapers; GC: collector-gatherers; FC: collector-filters; SH: shredders; OM: omnivores. The W is the whole study area, the T is upstream tributary, the H is upper main stream, and the M is middle stream

Fifty species of macrobenthos were collected, including 32 species of PRs, 7 species of SCs, 3 species of GCs, 4 species of FCs, 3 species of SHs, and 1 species of omnivore, which accounted for 64%, 14%, 6%, 8%, 6%, and 2% of the total species, respectively. Among these groups, OMs were absent from the upstream tributary habitats while the feeding functional groups were fully represented in the other two habitat types. Across the whole study area, the percentage of PRs was the highest (64%) while that of OMs was the lowest (only 2%). The composition of the FFGs showed significant differences in the different habitats, with the upstream and midstream habitats being the most similar (Fig. 6). In terms of the density of the different FFGs, the density of PRs reached the maximum value (1251 ind./m^2^, accounting for 41.81% of the total density) across the whole region and represented the largest proportion in all river reaches, with a value ranging from 263 to 677 ind./m^2^ (the proportion ranged from 34.79 to 60.98%). The highest value was observed in the middle stream, and the lowest value was observed in the upper mainstream. In addition, the density of SCs reached a maximum (628 ind./m^2^) in the middle stream. In terms of biomass, SCs reached the maximum value across the whole study area (92.85 g/m^2^, 50.58%) and the highest value was observed in the middle stream (79.7855 g/m^2^).

### Biodiversity of macrobenthos

**Fig. 7:**
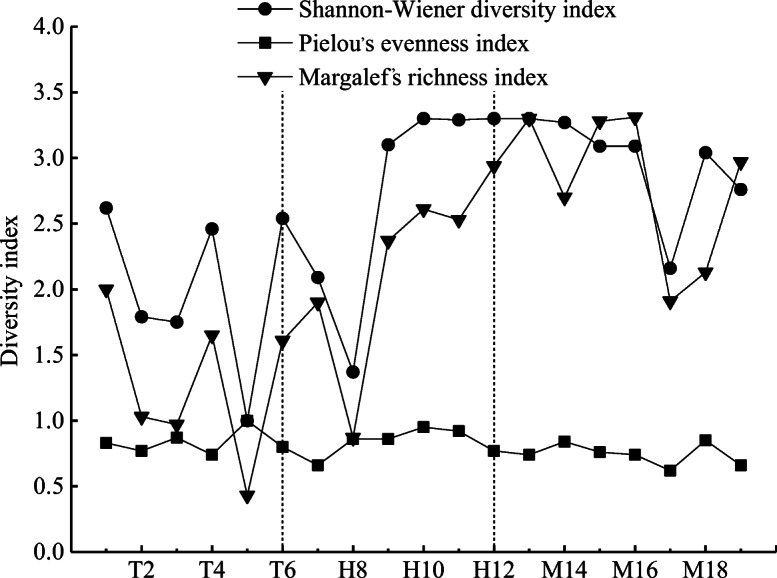
Variation trend of macrobenthic diversity index

The macrobenthos biodiversity showed relatively consistent trends from the upstream to midstream in terms of the *H*′, *d*_M_, and *J* (Fig. 7). As the altitude decreased, the *H*′ and *d*_M_ increased accordingly, with a large range of variation (varied from 1.00 to 3.30 and 0.43 to 3.31, respectively), and the trend in the fluctuation of the two indexes was consistent and similar to that for *S* (Fig. 2). It was confirmed that there was a correlation between the diversity indexes for the macrobenthos and species composition and density. Furthermore, the overall trend of *J* was relatively stable, with a relatively small range of variation (0.62 to 1.00). The mean values of the *H*′, *d*_M_, and *J* were 2.03, 2.74, and 2.96 for the upstream tributary, 1.28, 2.20, and 2.80 for the upper mainstream, and 0.84, 0.84, and 0.74 for the middle stream, respectively, showing that the *H*′ and *d*_M_ values for the middle stream were higher than those for the upstream tributary and main stream and that the values for the upper mainstream were also higher than those for the upstream tributary. These results indicated that the complexity and stability of the macrobenthic community in the middle stream were higher than those in the upper mainstream and that those in the upper mainstream were higher than those in the upper tributary. ANOVA was used to analyze the spatial distribution characteristics of the *H*′ and *d*_M_ values, and the results showed significant differences between the upper tributary and upper mainstream and between the middle stream and the upstream tributary (*P* < 0.05), while the difference between the upper main stream and the middle stream was not significant (*P* > 0.05). Pearson correlation analysis showed that the *H*′ was significantly positively correlated with DO, TN, and BOD_5_ (*P* < 0.05), with correlation coefficients of 0.474, 0.521, and 0.548, respectively. A significantly positive correlation was observed between *d*_M_ and WT, TN, and BOD_5_ (*P* < 0.05), with correlation coefficients of 0.476, 0.640, and 0.590, respectively. However, *J* was significantly negatively correlated with WT, EC, TDS, salinity, and TN (*P* < 0.05), with correlation coefficients of − 0.552, − 0.551, − 0.554, − 0.561, and − 0.478, respectively (Table 6).

**Table 6:** Correlation matrix analysis of macrobenthic assemblages and environmental parameters. *Indicating significant correlation at 0.05 level (*p* < 0.05); **indicating significant correlation at 0.01 level (*p* < 0.001). *WT*: water temperature; pH, *EC*: electrical conductivity; *TDS*: total dissolved particle; *DO*: dissolved oxygen; salinity; *TP*: total phosphorus; *TN*: total nitrogen; *NH*_*3-*_N: ammonia nitrogen; *BOD*_*5*_: biochemical oxygen demand; *COD*_*cr*_: chemical oxygen demand; *COD*_*Mn*_: potassium permanganate index. *H*′: Shannon-Wiener diversity index; *J*: Pielou’s evenness index; *d*_*M*_: Margalef’s richness index

Factors	WT	pH value	EC	TDS	DO	Salinity	TP	TN	NH_3_-N	BOD_5_	COD_cr_	COD_Mn_
Species	0.501*	0.220	0.122	0.114	0.514*	0.113	0.039	0.643*	0.034	0.631*	− 0.240	− 0.004
Density	0.419	0.211	0.269	0.270	0.611**	0.264	0.042	0.531*	0.124	0.500*	− 0.004	0.025
Biomass	0.572*	0.307	0.374	0.364	0.577**	0.368	0.024	0.571*	0.198	0.533*	0.047	− 0.221
*H*′	0.330	0.132	− 0.169	− 0.172	0.474*	− 0.179	0.046	0.521*	− 0.177	0.548*	− 0.384	0.027
*J*	− 0.552*	0.167	− 0.551*	− 0.554*	− 0.015	− 0.561*	− 0.018	− 0.478*	− 0.227	− 0.219	0.185	0.263
*d*_M_	0.476*	0.167	0.011	0.003	0.445	0.000	0.061	0.640**	− 0.022	0.590**	− 0.317	0.005

### Relationship between macrobenthos assemblages and environmental parameters

**Fig. 8:**
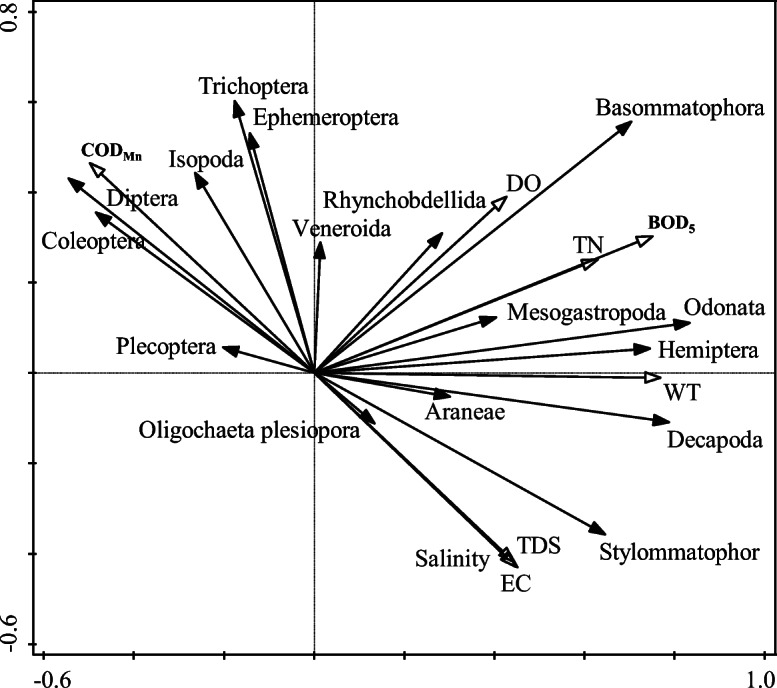
RDA sequence of macrobenthos and environmental parameters

The results of the DCA showed that the gradient length (SD) of the first ordination axis was the longest, with a value of 2.53 (SD < 3). Therefore, the linear-model RDA was the most appropriate for analyzing the relationships between the macrobenthic assemblages and environmental parameters (Fig. 8). The quadrant containing an arrow in the figure indicates whether a positive or negative correlation occurred between an environmental factor and the ordination axis. The correlation degree between an environmental factor and the community distribution is shown by the length of the arrow. The correlation between a certain environmental factor and the ordination axis is represented by the angle between the arrow and the sorting axis, with smaller angles indicating a greater correlation.

The RDA results showed that 78.3% of the species change information was explained by the selected environmental parameters. The eigenvalues of the first two ordination axes were 0.3401 and 0.1305, with these axes accounting for 13.05% and 10.28% of the variation in the macrobenthic community. The correlation coefficients between the species and environmental factors were as high as 0.9738 and 0.8370, and they indicated that the cumulative percentages of species and environmental factors along the first axis were 16.67% and 13.13%, respectively; thus, this axis had the largest contribution percentage of 43.42%. The total cumulative percentage of the relationship between species and environmental factors was as high as 82.01%, indicating that the relationships between species and environmental parameters could be best reflected by the ordination map (Table 7). According to the Monte Carlo replacement test, the main environmental variables that best explained the community structure of the macrobenthos in the different zones were BOD_5_, WT, TN, salinity, EC, TDS, and COD_Mn_ (*P* < 0.05) as shown in Table 8. Among these variables, BOD_5_ and WT had the largest marginal effects and accounted for 22.7% and 20.9% of the total (*P* < 0.05), indicating that these environmental variables were the key factors affecting the characteristics of the macrobenthic community (*F* = 5.0, *P* = 0.002; *F* = 4.5, *P* = 0.004), followed by TN (*F* = 3.2, *P* = 0.002), with an interpretation rate of 15.7% (*P* < 0.05), and salinity, EC, TDS, and COD_Mn_ (*F* = 2.5, *P* = 0.018; *F* = 2.4, *P* = 0.034; *F* = 2.4, *P* = 0.038; *F* = 2.3, *P* = 0.038, respectively). However, significant correlations were not observed between the other water environmental factors and the macrobenthic community (*P* > 0.05).

**Table 7:** RDA analysis of macrobenthic community and environmental parameters

Item	Axis1	Axis2	Axis3	Axis4
Eigenvalue	0.3401	0.1305	0.1028	0.0689
Species-environment correlation	0.9738	0.8370	0.9480	0.9344
Cumulative percentage of species data variance	34.01	47.06	57.34	64.23
Cumulative percentage of species-environment variance	43.42	60.09	73.22	82.01

**Table 8:** Monte Carlo test results of macrobenthic community and environmental parameters. *Indicating significant correlation at 0.05 level (*p* < 0.05). *WT*: water temperature; pH, *EC*: electrical conductivity; *TDS*: total dissolved particle; *DO*: dissolved oxygen; salinity; *TP*: total phosphorus; *TN*: total nitrogen; *NH*_*3-*_*N*: ammonia nitrogen; *BOD*_*5*_: biochemical oxygen demand; *COD*_*cr*_: chemical oxygen demand; *COD*_*Mn*_: potassium permanganate index

Parameters	Interpretation rate (%)	*F*	*P*	Parameters	Interpretation rate (%)	*F*	*P*
BOD_5_	22.7	5.0	0.002*	COD_Mn_	11.8	2.3	0.038*
WT	20.9	4.5	0.004*	pH value	10.6	2.0	0.064
TN	15.7	3.2	0.002*	DO	9.1	1.7	0.120
Salinity	12.6	2.5	0.018*	COD_cr_	2.6	0.5	0.896
EC	12.3	2.4	0.034*	NH_3-_N	1.9	0.3	0.972
TDS	12.2	2.4	0.038*	TP	1.8	0.3	0.954

The response relationship between the characteristics of the macrobenthic organisms and environmental indicators was explored based on the above discussion and combined with the results of the Pearson correlation matrix analysis (Table 6). Significant positive correlations were observed between the number, density, and biomass of the macrobenthic species and the DO, TN, and BOD_5_ (*P* < 0.05). In addition, the number and biomass of the species and WT also showed significant positive correlations (*P* < 0.05). The distribution of the macrobenthic community was related to multiple environmental conditions in the upper and middle reaches of the HRB, which were relatively complex.

## Discussion

### Ecological characteristics of the macrobenthos assemblages in different zones

The species composition and community structure of macrobenthos assemblages are directly affected by the nutrient concentration, hydrodynamic conditions, aquatic organism foraging pressure, and hydrological dynamics (Tews et al. 2004; Reynolds 2006; Shostell and Williams 2007). In addition, dynamic changes in time and geographic location cannot be ignored (Stomp et al. 2011). The Heihe River originates from the northern foot of the Qilian Mountains. Due to the differences in forest coverage, topography, and geology in the different sections of the river, the habitat conditions are complex and diverse and thus provide abundant living conditions for species with different tolerance levels. The results showed that the spatial divergence of the macrobenthic community was extremely significant from the upper reaches to the middle reaches regardless of the species composition or existing stock of macrobenthic fauna, which were closely related to the climate, geographical characteristics, and pollution status of the HRB. The middle stream region maintained a high diversity of macrobenthos, and the abundance of species and the standing stock were higher than that in the upstream tributary and mainstream. Compared with the upper reaches, the middle reaches were greatly affected by anthropogenic activities, which led to complex and changeable river habitat conditions; in addition, the nutrients were abundant and the stability and heterogeneity of the riverbed were high. Previous studies have shown that greater riverbed sediment stability and habitat heterogeneity are associated with high biodiversity (Shumway et al. 2007; Pandey and Thiruchitrambalam 2019).

Biodiversity, as an objective index, is used to measure the abundance of biological resources in a region. In this study, the macrobenthos diversity in the upstream tributary was significantly lower than that in the upper mainstream and middle stream. The macrobenthos community showed not only a simple structure and poor stability but also a weak ability to resist external environmental changes and internal population fluctuations. These findings are primarily because the community is influenced by the upstream tributary located in the Qilian Mountains in the northeast of the Qinghai Tibet Plateau, which has a high altitude (2783 m on average) and low temperature, and the annual accumulated temperature of the river water is affected by the incorporation of ice and snow meltwater, which has a relatively low temperature (the annual average temperature is less than 2 °C). In addition, the large slope of the riverbed and flow velocity have a great influence on the survival and reproduction of macrobenthos organisms and are suitable for the survival of flowing-water type and narrow cold-water type species; moreover, such conditions also provide an appropriate habitat for certain PRs, such as Plecoptera and Trichoptera, which are adapted to the riparian habitat (Allen et al. 2002; Burford et al. 2007; Dixon et al. 2009). PRs were the main FFG in the study area. The diversity of species was also affected by the increase in longitude, latitude and altitude (Jacobsen et al. 1997). In addition, the river section was less subject to anthropogenic activities, and it showed a lack of nutrients, stability of the riverbed bottom, and simple habitat heterogeneity (Li et al. 2000; Li et al. 2015a), resulting in low biodiversity, which is relatively in line with the characteristics of inland river systems.

Compared with the middle stream, the upstream area of the main stream showed relatively scarce macrobenthos organisms. In addition, this area had a more complex and stable community structure and the community also showed strong resistance to external environmental changes and internal population fluctuations. Because the upstream tributaries (Yeniugou River and Babao River) converge, they extend to the main stream and are polluted by point sources and nonpoint sources. Along the river course, the heterogeneity of the river habitats is often affected by organic matter enrichment from domestic effluents and waste from livestock breeding and industrial and mining enterprises, which are directly or indirectly discharged into the river channel, thereby increasing the input of nutrients. Moreover, the river flows through mountain and valley regions, which host fewer pollution sources. However, due to the influence of hydrodynamic regulation, the original hydrodynamic conditions and dynamic balance of material transport have been destroyed by dam interception while the organic matter discharged from external pollution sources in the upstream water accumulates along the river. Additionally, the construction of gates and dams has disrupted the continuity of the river and hindered the natural migration of aquatic organisms, resulting in a sharp reduction in species diversity and even the disappearance of some organisms (Pringle et al. 2000; Dudgeon et al. 2006; Carlisle et al. 2011). Furthermore, the food sources for the macrobenthos were influenced by sand and gravel mining, which changed the physicochemical properties of the river, and the subsequent turbid water quality affected photosynthesis by primary producers. The standing stock and diversity of the macrobenthos were also directly affected, which directly changed the structure of the riverbed and damaged the habitat environment (Nairn et al. 2004; Erftemeijer and Lewis 2006).

The biodiversity of the macrobenthos was relatively high in the middle stream, where the community structure was relatively complex and showed gradually increasing stability as well as strong resistance to external environmental changes and internal population fluctuations. The river section is located in the plain area of the Hexi Corridor, with a high intensity of human activities, such as the discharge of agricultural irrigation and industrial and domestic sewage into the river, and the organic matter and nutrient content in the water body both increase due to the continuous input and accumulation of exogenous substances. This section provided abundant sources of food and complex habitats for macrobenthos with different living habits, among which the pollutant-sensitive groups gradually decreased in abundance while the pollution-tolerant groups gradually increased in abundance (Covich et al. 2004; Soetaert 2015). Additionally, the water flow slowed and the WT and transparency improved in the midstream region. Aquatic vascular plants that occur along the river course not only accumulate organic debris, stabilize riverbed sediments, and promote aquatic organisms but also provide abundant nutrients and suitable habitats for macrobenthos (Li et al. 2000; Devine and Vanni 2002; Hao et al. 2014; Soetaert 2015), such as GCs (Oligochaeta), FCs and SCs (Mollusca), which rely on water flow to obtain food (Fu et al. 2008; Jiang et al. 2009). The individual biomass of mollusks was relatively large, making them absolutely dominant over arthropods, and they mainly inhabited the sediment in the shallow water area of the riverbed, which was rich in organic matter. In the slow-flow water environment with lush aquatic plants, the species richness and population number of mollusks tended to increase (Cai et al. 2009). The suitable environmental types and complex habitats maintained the abundance of macrobenthos and the stability of the ecosystem.

### Relationships with environmental parameters in different zones

Environmental factors in water bodies, such as the nitrogen and phosphorus concentration, DO, EC, and WT, have been reported to directly affect the composition, life cycle, and distribution of macrobenthic communities (Miserendino 2001; Yan et al. 2005; Cooper et al. 2007; De Jonge et al. 2009), with the WT considered the key natural variable affecting the growth of macrobenthos. Nutrients are regarded as important chemical indicators that affect macrobenthos survival, and abundant nutrients often cause protozoa to proliferate in large numbers (Cooper et al. 2007). A previous evaluation of water quality in the HRB found that the best water quality occurred in the upstream reach while pollution occurred in the middle and lower reaches due to the discharge of industrial, agricultural and domestic sewage (Wang et al. 2019). This result was confirmed in this study. The results of the Pearson correlation analysis showed that the number of macrobenthos species and the standing stock were significantly correlated with the TN concentration in the upper and middle reaches of the HRB (*P* < 0.05). Combined with the results of the RDA, we also found that the TN concentration and the dominant groups (Odonata, Hemiptera, Decapoda, etc.) were significantly positively correlated. Additionally, BOD_5_, WT, salinity, EC, TDS, COD_Mn_, and DO were key environmental factors affecting the distribution of the community. The ecological attributes of the macrobenthos communities were closely related to the physicochemical characteristics of the water environment. The water supply in the HRB mainly originates from ice and snow meltwater from the Qilian Mountains. From upstream to downstream, the river crosses different climatic zones to form a unique ecological system. Only the Zhangye area includes three ecological types: the upstream Qilian Mountain area, the middle stream oasis agricultural area, and the downstream saline-alkali land desert area. Therefore, the divergence of water environmental factors under the different geographical patterns remains the key factor affecting the macrobenthic organisms, even on a relatively small spatiotemporal scale. The results of the RDA showed that the marginal effect of the single environmental factor WT was as high as 20.9% for the macrobenthic organisms in the upper and middle HRB, and the dominant groups of Mollusca, including *Suecinea* sp., *Cyraulus albus*, and *Radix auricularia*, were relatively concentrated in the middle stream. Cooper et al. (2007) showed that WT was a key factor affecting increases and decreases in the density and population distribution of aquatic insects. Because the HRB is an inland river system with an overall low WT, *Chironomid* sp. were rarely collected in aquatic insects. In addition, increasing temperatures within the appropriate temperature range can accelerate the growth of macrobenthos, while decreasing temperatures during cold months lead the growth rate of some species to slow or even stop. *Chironomid larvae* can reproduce from spring to autumn and grow rapidly under high-temperature conditions in summer. However, with the continuous decrease in temperature leading up to the cold winter, their growth rate slows or even stops completely (Pringle et al. 2000; Dudgeon et al. 2006). Changes in temperature have an important effect on the survival of macrobenthos and also affect the concentration of other physicochemical indicators in water. The Pearson correlation analysis showed that there were strong correlations between WT and EC, TDS, salinity, TN, and COD_Mn_.

Yan et al. (2005) showed that the diversity of macrobenthic species was negatively correlated with the concentration of nutrients in the water body. Nutrients exacerbate the eutrophication of the water body, and if the concentration is too high, the DO concentration in the bottom water environment will decrease, which will increase the sulfide content in the sediment bottom and water body; these changes restrict the distribution of sensitive species and even lead to the disappearance of some species (Keeley et al. 2014). Wang et al. (2016) found that DO was extremely important in the growth and development of macrobenthos species, especially when photosynthesis essentially stopped at night and the oxygen demand was insufficient to support their survival. The BOD_5_ and COD_Mn_ values indicate that the water body is polluted by organic oxygen-consuming substances, such as industrial, agricultural, and domestic sewage, and high concentrations of such substances correspond with more severe pollution of the water body, which affects the distribution of fish, macrobenthos, and other aquatic organisms (Cooper et al. 2007; Peng et al. 2014; Li et al. 2015b). In the midstream of the HRB, the discharge of a large number of organic pollutants and nutrients, such as from enterprise operations, animal husbandry, and domestic sewage, and the inflow of the surrounding tributaries greatly threaten the ecological environment of the river, where some sensitive organisms adapted to the shortage of anoxic environments gradually decreased or even disappeared. This result indicated that the pollution-tolerant groups gradually increased while the sensitive groups gradually decreased, leading to a homogenous community structure. In addition, the smaller pollution-tolerant species will gradually replace the larger species and eventually become dominant (Devine and Vanni 2002; Keeley et al. 2014). The midstream had the most pollution-tolerant species (19 species) and moderately pollution-tolerant species (15 species) and only 3 sensitive species. The spatial complexity and heterogeneity of the water environment was subject to different degrees of human interference, thereby restricting the survival of different groups of organisms and resulting in the spatial divergence of the species composition and diversity.

The unique geographical location and hydrological characteristics of the HRB and the results of this analysis indicate that the WT was generally low in this area and the distribution of macrobenthic faunal was closely related to the WT, other natural factors, and external pollutant and nutrient inputs. These findings indicated that natural factors and human activities were the dual driving factors restricting the structure and diversity of the macrobenthos community. In turn, we discussed how to improve the ecological environment and comprehensively manipulate the environment of the basin based on the influencing factors to maintain the dynamic balance of the ecosystem in the HRB.

## Conclusions

1. A total of 50 macrobenthic species were identified in this survey, among which arthropods were absolutely dominant (74%). The community structure showed significant spatial heterogeneity, with the highest species abundance (74%) in the midstream reach, where the dominant species were mostly pollution-tolerant arthropods and moderately tolerant mollusks. The next highest abundance was observed in the upstream mainstream (54%), where moderately tolerant arthropods and pollution-tolerant mollusks were the dominant species. The upstream tributary had the lowest relative abundance (44%), with sensitive and moderately tolerant arthropods being the dominant species.
2. The results of the RDA method combined with the Pearson correlation analysis results showed that the BOD_5_, WT, TN, salinity, EC, TDS, DO, and COD_Mn_ (*P* < 0.05) were the vital factors affecting the spatial dynamics of the macrobenthic assemblages in the upper and middle reaches of the HRB.
3. The spatial distribution characteristics of the macrobenthic assemblages were closely related to the physicochemical properties of the water body. Maintaining habitat complexity and good water quality is key to preserving the diversity and stability of species.
